# Targeting pathophysiological changes using biomaterials-based drug delivery systems: A key to managing inflammatory bowel disease

**DOI:** 10.3389/fphar.2022.1045575

**Published:** 2022-11-10

**Authors:** Sahar Mohajeri, Saeed Moayedi, Shabnam Mohajeri, Abbas Yadegar, Ismaeil Haririan

**Affiliations:** ^1^ Department of Pharmaceutical Biomaterials, Faculty of Pharmacy, Tehran University of Medical Sciences, Tehran, Iran; ^2^ Faculty of Food Science and Technology, Gorgan University of Agricultural Sciences and Natural Resources, Gorgan, Iran; ^3^ Foodborne and Waterborne Diseases Research Center, Research Institute for Gastroenterology and Liver Diseases, Shahid Beheshti University of Medical Sciences, Tehran, Iran

**Keywords:** pathophysiological changes, biomaterials, drug delivery systems, inflammatory bowel disease, colon

## Abstract

Inflammatory bowel disease (IBD) is a gastrointestinal disorder, affecting about several million people worldwide. Current treatments fail to adequately control some clinical symptoms in IBD patients, which can adversely impact the patient’s quality of life. Hence, the development of new treatments for IBD is needed. Due to their unique properties such as biocompatibility and sustained release of a drug, biomaterials-based drug delivery systems can be regarded as promising candidates for IBD treatment. It is noteworthy that considering the pathophysiological changes occurred in the gastrointestinal tract of IBD patients, especially changes in pH, surface charge, the concentration of reactive oxygen species, and the expression of some biomolecules at the inflamed colon, can help in the rational design of biomaterials-based drug delivery systems for efficient management of IBD. Here, we discuss about targeting these pathophysiological changes using biomaterials-based drug delivery systems, which can provide important clues to establish a strategic roadmap for future studies.

## Introduction

IBD is a group of idiopathic, chronic inflammatory disorders of the gastrointestinal tract which includes two major forms, ulcerative colitis (UC) and Crohn’s disease (CD) (Windsor and Kaplan, 2019; Bisgaard et al., 2022; Plevris and Lees, 2022). It is renowned that chronic inflammation in intestinal tract is an important feature of both UC and CD. They are complex diseases and the basic pathological process looks to be a combination of immunologic disturbances and genetic predisposition. In other words, a dysregulated immune reaction to a normal or altered gut microbiome in genetically prone individuals plays an important role in causing this disease (Cosnes et al., 2011; Khor et al., 2011; Molodecky et al., 2012; Dal Buono et al., 2021; Jeffery et al., 2022). It is activated by the interaction of environmental factors with the autoantigens supposed to reside on nonpathogenic commensal bacteria of gut microbiota (Loftus Jr, 2004; Zhang et al., 2021; Zhuang et al., 2021).

Multiple environmental agents, involved in the development of IBD, include factors like infectious pathogens, diet, and autoantigens residing in the gut microbiome (Ananthakrishnan, 2015; Kaplan, 2015; Raoul et al., 2022). It is assumed that there is a relation between raised use of animal meat and refined sugars and the risk of IBD development. The mentioned dietary components seem to interact with intestinal flora, leading to the production of pro-inflammatory factors. Persons who use less dietary fiber, raw fruits, and vegetables are more at risk for IBD (Asakura et al., 2008; Sasson et al., 2021; Sugihara and Kamada, 2021).

At present, existing therapies can’t adequately control clinical symptoms in a significant number of IBD patients. Consequently, novel treatments for IBD is needed (Ma et al., 2019; Camba-Gómez et al., 2021). It is worth noting that considering the pathophysiological changes occurred in the gastrointestinal tract of the patients with IBD can help in the rational design of new treatments for effective management of IBD (Nakase, 2020). Recently, the attention of researchers has been attracted to the development of drug delivery systems as a promising strategy for the efficient management of IBD. Until now, some review articles were published regarding the treatment of IBD based on drug delivery systems (Wang et al., 2022). However, there is a need for a review focused on targeting pathophysiological changes *via* biomaterials-based drug delivery systems (biomaterials-based DDSs) that can provide key clues to establish a strategic roadmap for future studies.

## Conventional therapies for IBD and the importance of biomaterials-based DDSs

Currently, there is no definitive method for the medical cure of IBD. The management of this disease engages the utilization of anti-inflammatory drugs which can considerably decrease the symptoms of disease and help preserve its remission. Drugs employed to treat the signs of IBD consist of aminosalicylates (like sulfasalazine and Mesalamine), corticosteroids, immunomodulators (such as azathioprine and methotrexate), Tofacitinib (a Janus kinase inhibitor), TNF-α blocking biological agents (like infliximab and adalimumab), and anti-integrin biological factors (such as natalizumab) (Fakhoury et al., 2014; Al-Bawardy et al., 2021; Battat and Sandborn, 2021; Burr et al., 2021; Dudek et al., 2021; Hanzel et al., 2022). For the patients who do not respond to less aggressive treatment like sulfasalazine and azathioprine, biological drugs such as infliximab, adalimumab, and natalizumab are utilized. However, biological drugs have a high cost and increase the considerable risk of developing infections. Corticosteroids are anti-inflammatory drugs with good efficiency in some inflammatory diseases, but long-term utilization of corticosteroids to treat both UC and CD is not successful and can lead to severe undesirable effects (Koda-Kimble, 2012; Danese et al., 2020; Bruscoli et al., 2021). From a therapeutic point of view, pharmacokinetic considerations (including absorption, distribution, metabolism, and elimination of drugs) affect the probability of therapeutic success. Oral, rectal, and intravenous administrations are the most common routes of drug administration for IBD treatment. Due to higher patient compliance than injections and lower production costs, oral drug administration is considered one of the most convenient routes. Nonetheless, oral administration of drugs has some restrictions such as low absorption of hydrophobic drugs, delivery to normal tissues, the probability of degradation during passage *via* the gastrointestinal tract, and hepatic first-pass metabolism following the intestinal absorption process. The most important benefit of rectal administration (especially *via* enema and suppository) is topical drug delivery to the injured colon. However, maintaining the enema or suppository for long periods of time in colon is the main limitation of rectal administration. Intravenously administered drugs do not undergo hepatic first-pass metabolism, which it is the main advantage of intravenous use. Despite this benefit, intravenous administration has some challenges like nonspecific distribution in normal tissues and short blood circulation time (Derijks et al., 2018; Patel et al., 2020; Denesh et al., 2021). Hence, the modulation of pharmacokinetic properties of drugs to improve the targeted treatment and decrease undesirable side effects could be a game-changer in the efficient treatment of IBD. Due to their unique properties, especially active functional groups present in biomaterials structure, modification of biomaterials-based DDSs can help in the modulation of pharmacokinetic properties of drugs. Among biomaterials used in developing new targeted biomaterials-based DDSs to treat IBD, biomaterials based on polymer (such as poly (ethylene glycol) (PEG), poly (lactic acid) (PLA), poly (lactic-co-glycolic acid) (PLGA), hyaluronic acid, and chitosan) and biomaterials based on lipids (like phospholipids) have attracted the most attention of researchers (Wang et al., 2022).

## Pathophysiological changes in the gastrointestinal tract of IBD patients

The most important pathophysiological changes in the gastrointestinal tract of the patients with IBD include changes in pH, surface charge, the concentration of reactive oxygen species (ROS), and the expression of some biomolecules at the inflamed colon. In normal physiological conditions, colonic luminal pH is in the range of 7-8, which is affected by many factors especially bicarbonate and lactate production (Hall and Hall, 2020). Colonic luminal pH is lower than normal in the patients with IBD, mainly because of changes in bicarbonate and lactate production (Zhang et al., 2017). Until now, some studies were performed to evaluate accurate colonic pH value in IBD patients, but similar results were not found. However, most studies have confirmed that colonic pH in the patients with IBD is relatively acidic (Cummins et al., 2019).

**FIGURE 1:**
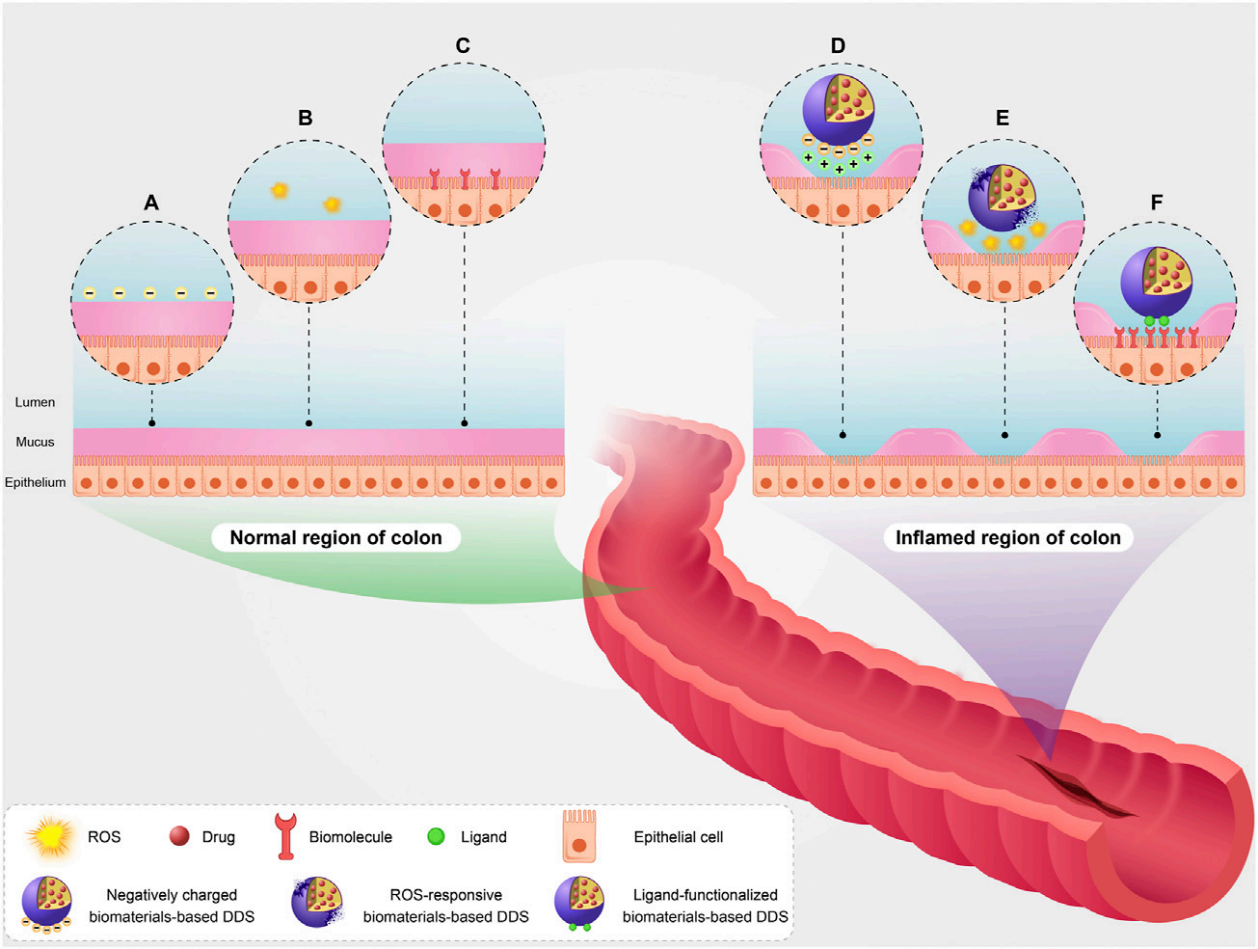
A schematic illustration of targeting pathophysiological changes using biomaterials-based drug delivery system (biomaterials-based DDS) in the colon of patients with IBD. Surface charge of epithelium **(A)**, basic ROS level **(B)**, and basic expression level of some biomolecules such as inflammatory biomarkers **(C)** in the normal region of colon. Targeting pathophysiological changes including positive surface charge of injured epithelium using negatively charged biomaterials-based DDS **(D)**, high ROS level using ROS-responsive biomaterials-based DDS **(E)**, and overexpression of the biomolecules using ligand-functionalized biomaterials-based DDS **(F)** in the inflamed region of colon.

Another important pathophysiological factor in the colon of IBD patients is surface charge. In normal physiological conditions, surface charge of colon epithelium is negative (Figure 1A). Mucosal damage and positively charged proteins accumulation such as transferrin are among the most important pathophysiological changes in the inflamed colon. This results in the formation of positive charges at the injured epithelial surface (Figure 1D) (Ramasundara et al., 2009; Tirosh et al., 2009; Kesharwani et al., 2018). Oxidative stress plays a key role in the pathogenesis and progression of IBD. Persistent oxidative stress and increased production of ROS can lead to local tissue damage and inflammation. In normal physiological conditions, ROS concentration is low in the colon epithelium (Figure 1B). In the patients with IBD, the ROS concentration is high in the inflamed regions of the colon (Figure 1E) (Aviello and Knaus, 2018; Guan and Lan, 2018; Hu et al., 2019; Bertoni et al., 2020; Dziąbowska-Grabias et al., 2021).

The expression of some biomolecules like inflammatory biomarkers in the injured colon is increased during IBD development compared with their basic expression level in normal physiological conditions (Figures 1C,F). The overexpression of glycoprotein CD98, CD44, and folate receptor is among the most important pathophysiological changes in the injured colon (Naserifar et al., 2020; Brazil and Parkos, 2022; Rahiman et al., 2022). On the other hand, it is noteworthy that expression of some biomolecules on the surface of capillary endothelial cells raises in the inflamed colon. The overexpression of intercellular adhesion molecule-1 (ICAM-1), vascular cell adhesion molecule-1 (VCAM-1), P-Selectin, and E-Selectin is among the most important pathophysiological changes on the surface of capillary endothelial cells in damaged regions of the colon (Zhao et al., 2019; Bui et al., 2020; Schmid et al., 2022).

## Targeting pathophysiological changes using biomaterials-based DDSs

Considering the pathophysiological changes which occurred in the gastrointestinal tract due to IBD can help in the rational design of biomaterials-based DDSs for efficient management of IBD. In particular, changes in the inflamed regions of the colon can be a good clue to specifically targeting the inflamed area rather than the whole colon. A summary of studies on targeting pathophysiological changes using biomaterials-based DDSs is presented in Table 1.

**TABLE 1:** Summary of studies on targeting pathophysiological changes using biomaterials-based drug delivery system (biomaterials-based DDS).

Targeted changes	Biomaterials-based DDS	Drug/active agent	Study model	References
pH	Aminoclay-based nanocomplex coated with Eudragit S100	Infliximab	*in vitro* and *in vivo*, oral administration, DSS-induced colitis mice	Lee et al. (2022)
	Pectin-PEG-methacrylic acid hydrogel	Sulfasalazine	*in vivo*, oral administration, DSS-induced colitis rat	Abbasi et al. (2019)
	Eudragit FS30D- PLGA nanoparticles	Cyclosporine A	*in vitro* and *in vivo*, oral administration, DSS-induced colitis mice	Naeem et al. (2018)
Positive surface charge	1,2-distearoyl-sn-glycero-3-[phospho-rac-(1-glycerol)-based liposomes	-	*in vivo,* oral administration, dinitrobenzensulfonic acid-induced colitis rat	Jubeh et al. (2004)
	Cerium-metal-organic framework@poly (sodium-4-styrenesulfonate)	5-aminosalicylic acid	*in vivo,* rectal administration, trinitrobenzenesulfonic acid-induced colitis mice	Yin et al. (2021)
	Heparin hydrogel	Heparin, silver	*ex vivo* and *in vivo,* rectal administration, DSS-induced colitis mice	Hong et al. (2022)
	Ascorbyl palmitate hydrogel	Dexamethasone	*in vivo,* rectal administration, DSS-induced colitis mice	Zhang et al. (2015)
High concentration of ROS	Poly [(*N*-acryloylmorpholine)-*b*-(*N*-acryloylthiomorpholine)] micelles (ROS-responsive bond: thioether)	-	*in vitro*	Gardey et al. (2022)
	Phenylboronic ester-carboxylmethyl chitosan micelles (ROS-responsive bond: ester)	Berberine	*in vitro* and *in vivo,* oral administration, DSS-induced colitis mice	Zhao et al. (2021)
	4-(hydroxymethyl) phenylboronic acid pinacol ester-β-cyclodextrin nanoparticles (ROS-responsive bond: ester)	Genistein	*in vivo,* oral administration, DSS-induced colitis mice	Fan et al. (2021)
	4-(hydroxymethyl) phenylboronic acid pinacol ester-β-cyclodextrin nanoparticles (ROS-responsive bond: ester)	Tempol	*in vivo,* oral administration, DSS-induced colitis mice	Zhang et al. (2016)
	Poly-(1,4-phenyleneacetone dimethylene thioketal) nanoparticles (ROS-responsive bond: thioketal)	TNF-α-siRNA	*in vitro* and *in vivo*, oral administration, DSS-induced colitis mice	Wilson et al. (2010)
	4-(hydroxymethyl) phenylboronic acid pinacol ester-β-cyclodextrin nanoparticles (ROS-responsive bond: ester)	Ac2-26 peptide (derived from annexin A1)	*in vitro* and *in vivo*, oral administration, DSS-induced colitis mice	Li et al. (2019b)
Overexpression of biomolecules	CD98 Fab-functionalized PLA nanoparticles (targeting CD98)	-	*in vivo,* oral administration, DSS-induced colitis mice	Xiao et al. (2014)
	Hyaluronic acid nanoparticles (targeting CD44)	Lysine-Proline-Valine tripeptide	*in vivo,* oral administration, DSS-induced colitis mice	Xiao et al. (2017)
	Folate-functionalized PLGA/PLA nanoparticles (targeting folate receptor)	6-shogaol	*in vitro* and *in vivo,* oral administration, DSS-induced colitis mice	Zhang et al. (2018)
	Folate-functionalized poly (amidoamine) dendrimers (targeting folate receptor)	-	*in vivo,* intravenous injection-DSS-induced colitis mice	Poh et al. (2017)
	Sialyl-Lewis^x^-functionalized PLGA microspheres (targeting P-Selectin)	-	*in vitro*	Eniola et al. (2002)
	E-Selectin antibody-functionalized PLA–PEG microparticles (targeting E-Selectin)	-	*in vivo,* intravenous injection, TNF-α**-**treated mice	Sakhalkar et al. (2003)
	Neutrophils-simulated liposomes (targeting ICAM-1)	KGF	*in vitro* and *in vivo,* intravenous injection, DSS-induced colitis mice	Zhao et al. (2019)

### Targeting changes in pH

In IBD patients, colonic luminal pH is lower than normal. This important pathophysiological change can be used to efficiently deliver drugs to the injured colon by pH-responsive biomaterials-based DDSs. For example, pH-responsive aminoclay-based nanocomplex is one of the appropriate biomaterials-based DDSs for targeting changes in colonic luminal pH. Aminoclay is a biocompatible and water-soluble derivative of the highly disordered talc-like 2:1 trioctahedral magnesium phyllosilicates with covalently attached aminopropyl groups. Aminoclay has a positive charge and is an appropriate candidate to deliver drugs which have a positive charge at acidic pH, because electrostatic repulsion between aminoclay and positively charged drug leads to drug release from aminoclay in the inflamed colon. However, it is noteworthy that there is an important challenge facing the development of these systems. Acidic pH in stomach can result in drug release in the upper gastrointestinal tract (Lee et al., 2019). To overcome this challenge, an outer coating layer (insoluble in acidic conditions) in these systems is needed. Interestingly, Lee et al. developed pH-responsive nanocomplex based on aminoclay-infliximab coated with Eudragit S100. Infliximab is IgG monoclonal antibody that binds specifically to human TNF-α and is used to treat inflammatory disorders such as IBD. Eudragit S100 is a copolymer based on methacrylic acid and methyl methacrylate. Eudragit S100 is a pH-sensitive polymer that dissolves at a pH above 7.0, and therefore can minimize premature drug release in the upper gastrointestinal tract. Infliximab has a positive charge at pH below its isoelectric point (about 7.6), and thus electrostatic repulsion between aminoclay and infliximab results in drug release from the nanocomplex in the inflamed colon. The release of infliximab from the nanocomplex at pH 5.5 was confirmed by *in vitro* release study. The oral administration of this nanocomplex showed great accumulation of the nanocomplex as well as the release of infliximab in the injured colon of DSS-induced colitis mice. In addition, pro-inflammatory factors (including IL-6 and TNF-α) were decreased after the oral use of the nanocomplex (Lee et al., 2022).

In another study, the treatment of DSS-induced colitis rat with pH-responsive pectin-PEG-methacrylic acid hydrogel loaded with sulfasalazine showed excellent accumulation of the hydrogel as well as the release of sulfasalazine in the inflamed colon (Abbasi et al., 2019). Similarly, Naeem et al. developed pH-responsive cyclosporine A-loaded Eudragit FS30D- PLGA nanoparticles. Eudragit FS30D is a tripolymer comprising poly (methyl methacrylate, methyl acrylate, methacrylic acid), which is one of appropriate pH-sensitive biomaterials for colon delivery. The release of cyclosporine A from the nanoparticles at colonic pH was confirmed by *in vitro* release study. Furthormore, the results of this study showed an increase in the colon length and a reduction in Disease Activity Index (DAI) score after the oral use of the nanoparticles in mice with colitis induced by DSS, indicating the good functionality of the nanoparticles in the alleviation of colitis symptoms (Naeem et al., 2018). In general, targeting changes in pH *via* pH-responsive biomaterials-based DDSs can be an appropriate potential candidate to deliver potent drugs to the damaged colon. However, their efficacy and safety need to be further verified in more comprehensive studies.

### Targeting changes in surface charge

The formation of positive charges at the injured epithelial surface supplies a molecular target and anchor for biomaterials-based DDSs with negative surface charge (Figure 1D) (Jubeh et al., 2006; Li W. et al., 2018). Thus, negatively charged particles illustrate preferential attachment to the damaged areas of colon *via* electrostatic interaction with these proteins. For example, due to their unique properties (such as biocompatibility, excellent entrapment capacity, and amphiphilic nature), liposomes as one of biomaterials-based DDSs are promising candidates for the encapsulation of diverse drugs including both hydrophobic and hydrophilic compounds. Interestingly, negatively charged liposomes showed preferential adhesion to the inflamed colon of dinitrobenzenesulfonic acid-induced colitis rats *via* electrostatic interaction. They also had a two-fold higher cumulation in the inflamed regions compared with cationic and neutral liposomes, therfore negatively charged liposomes can be valuable to deliver anti-inflammatory drugs to the inflamed colon (Jubeh et al., 2004).

Conventional enemas are usually employed in mild-to-moderate colitis as a fundamental form of topical drug delivery to the injured colon. Nevertheless, in classic enema-based formulations, the patients may need to maintain the enema for prolonged periods of time which is hard when affected by diarrhea and fecal urgency. For this reason, enemas with the ability of local and specific targeting of inflamed tissue can be helpful to deliver anti-inflammatory drugs to the injured epithelial surface (Date et al., 2017; Date et al., 2018; Zhai et al., 2018; Ahmad et al., 2021). Metal-organic frameworks (MOFs) as another biomaterials-based DDSs are good candidates for drug delivery as well as cell targeting, mainly because of low toxicity and great drug loading capacity (Beg et al., 2017; Wang et al., 2018; Lawson et al., 2021). For example, negatively charged cerium-MOF@poly (sodium-4-styrenesulfonate)-based enema loaded with 5-aminosalicylic acid exhibited preferential attachment to the injured areas of the colon through electrostatic interaction in surgical specimens of IBD patients and colitis mice. This enema showed excellent therapeutic efficacy *via* diminishing inflammatory cytokines and repairing intestinal barrier function in comparison with the administration of free drug (Yin et al., 2021).

It is worth mentioning that hydrogel-based enema is one of the most attractive biomaterials-based DDSs for local drug delivery to the inflamed colon that not only has great properties like biocompatibility, sustained release of a drug, and high drug carrying capacity, but also aids in preventing the infection of injured epithelial tissue *via* the formation of a physical barrier (Garcia-del et al., 2020; Guo et al., 2021). Recently, Hong et al. developed enema based on negatively charged heparin-silver-bovine serum albumin hydrogel for local targeting the injured colon *ex vivo* and *in vivo* experiments. Heparin is a negatively charged biomaterial and has also anti-inflammatory activity. The hydrogel-based enema showed excellent accumulation in the injured colon compared with the healthy tissues as well as was effective in downregulation of interleukin-6 (a pro-inflammatory agent) in the colon’s inflamed regions, indicating the good functionality of this hydrogel for local targeting the damaged areas of the colon. Moreover, this enema exhibited the ability of accelerating mucosal healing *via* boosting Syndecan-1 (Sdc-1), a tissue repair agent, and can also help in the prophylaxis of inflamed colon infection *via* physical barrier formation (Hong et al., 2022). In a similar study, the treatment of colitic mice with enema based on negatively charged hydrogel microfibers loaded with dexamethasone showed preferential adhesion of these hydrogel microfibers to the inflamed regions of the colon compared to histologically normal tissues. These hydrogel microfibers were prepared using ascorbyl palmitate, a biomaterial which its safety was confirmed by the U.S. Food and Drug Administration (as generally recognized as safe). Due to its amphiphilic nature, ascorbyl palmitate is able to self-assemble into a hydrogel. Moreover, this study’s results indicated that the administration of this hydrogel-based system leads to a considerable decrease in inflammation and is related to lower serum concentrations of dexamethasone and consequently, less systemic side effects (Zhang et al., 2015).

In total, biomaterials-based DDSs with negative surface charge can adhere to the injured epithelial surface through electrostatic interaction and provide the local targeting of the inflamed colon. Therefore, these systems can be regarded as promising candidates for entry into the clinical trial phase. However, their efficacy and safety need to be further verified in more comprehensive studies.

### Targeting changes in the concentration of ROS

In IBD patients, the ROS concentration is high in the damaged areas of the colon. This important pathophysiological change can be used to efficiently deliver drugs to the injured colon by biomaterials-based DDSs (Figure 1E). For example, polymer micelles functionalized with ROS-responsive groups are one of the appropriate biomaterials-based DDSs for targeting changes in the concentration of ROS in inflamed regions of the colon. Gardey et al. developed ROS-sensitive polymer micelles based on amphiphilic block copolymers poly [(*N*-acryloylmorpholine)-*b*-(*N*-acryloylthiomorpholine)] through polymerization induced self-assembly. These micelles contain thioether groups in the hydrophobic core, which are susceptible to oxidation in the presence of ROS. The oxidation of thioether groups leads to the formation of hydrophilic sulfoxide groups and the degradation of micelles. Furthermore, the micelles were loaded with fluorescent dye Nile red to monitor the micelles degradation. When the micelles were incubated with monocytes isolated from patients with IBD, which had increased ROS production, the degradation of micelles was successfully occurred in comparison with the micelles incubated with monocytes isolated from healthy individuals (Gardey et al., 2022). In a similar research, the ROS-triggered release of berberine (anti-inflammatory agent) from ROS-responsive micelles based on phenylboronic esters-modified carboxylmethyl chitosan conjugated with berberine was confirmed by *in vitro* release study. Carboxymethyl chitosan is an interesting biomaterial for drug delivery because of their excellent properties such as the availability of functional groups for drug conjugation and biocompatibility. Furthermore, the results of this study demonstrated a reduction in Disease Activity Index (DAI) score and an increase in the colon length in mice with colitis induced by DSS, showing the great functionality of the micelles in the alleviation of colitis symptoms (Zhao et al., 2021).

Owing to their ROS-responsive properties, 4-(hydroxymethyl) phenylboronic acid pinacol ester and tempol moieties are valuable candidates for the conjugation to biomaterials for the preparation of ROS-responsive biomaterials-based DDSs (Li L. et al., 2018). In one study, the oral administration of ROS-responsive nanoparticles based on tempol-conjugated β-cyclodextrin loaded with genistein-4-(hydroxymethyl)phenylboronic acid pinacol ester showed great accumulation of the nanoparticles as well as the release of genistein in the inflamed colon of DSS-induced colitis mice. In addition, pro-inflammatory factors (including IL-1β and TNF-α) were decreased after the oral use of the nanoparticles. Genistein is a strong antioxidant factor that suppresses oxidative stress (Fan et al., 2021). Similarly, Zhang et al. synthesized ROS-responsive nanoparticles based on 4-(hydroxymethyl) phenylboronic acid pinacol ester-conjugated β-cyclodextrin loaded with tempol (as a superoxide dismutase-mimetic). Hydrogen peroxide results in hydrolysis of the nanoparticles and then this occurrence causes tempol release. The nanoparticles effectively accumulated in the injured regions of the colon in mice with DSS-induced colitis, thus noticeably decreasing the nonselective distribution after oral use. Moreover, the nanoparticles remarkably diminished symptoms related to colitis and considerably inhibited the expression of proinflammatory factors (including TNF-α, IL-1β, and INF-γ), with the effective performance compared with free tempol and a control drug carrier based on PLGA (Zhang et al., 2016).

In general, targeting changes in the concentration of ROS *via* biomaterials-based DDSs functionalized with ROS-sensitive groups can be a good potential candidate to deliver potent drugs to the inflamed colon. However, the following points should be considered before the clinical application of such ROS-sensitive systems. Until now, just the cytotoxicity phase of these ROS-responsive systems was investigated. Therefore, a comprehensive evaluation of the safety of these systems should be performed in several animal models because biocompatibility is an essential factor in the entry of new therapeutic systems into the clinical trial phase. Moreover, in normal concentrations of ROS, these systems should be inactive (Xu et al., 2016).

### Targeting changes in the expression of biomolecules

The expression of some biomolecules such as inflammatory biomarkers in the inflamed colon is increased during IBD development compared with their basic expression level in normal physiological conditions. Targeting these changes can be used to efficiently deliver drugs to the damaged areas of colon *via* biomaterials-based DDSs functionalized with a specific ligand in a way that, this ligand conjugated to biomaterials-based DDSs can recognize and bind to its specific complement biomolecule after reaching the intended tissue (Figure 1F). Consequently, it can lead to an increase in the accumulation of the drug in the inflamed areas of the colon. In the period of inflammation, the overexpression of inflammatory biomarkers is an important pathophysiological change (Liu et al., 2019; Busch et al., 2020; Yin et al., 2020). For example, the overexpression of glycoprotein CD98 is occurred in the inflamed colon (Guo et al., 2016; Rahiman et al., 2022). In a study, it was shown that the uptake of PLA-based nanoparticles conjugated to CD98 Fab is occurred by Colon-26 and RAW 264.7 cells *via* CD98-mediated endocytosis. In addition, these nanoparticles remarkably accumulated in the injured colon of mice with colitis induced by DSS compared to PEG-based nanoparticles (Xiao et al., 2014).

Another example of changes in the expression of biomolecules is the overexpression of CD44 in the inflamed colon (Lee et al., 2021; Lima et al., 2021; Brazil and Parkos, 2022; Kotla et al., 2022). CD44 is a principal cell surface receptor for hyaluronic acid, a main component present in the extracellular matrix. Hyaluronic acid is an anionic glycosaminoglycan distributed broadly in epithelial, connective, and neural tissues. It is considered a polymer composed of alternating monosaccharide units of N-acetylglucosamine and glucuronic acid. Moreover, it is worth mentioning that the negative surface charge of hyaluronic acid can help in improving its attachment to the injured colon. Therefore, drug deivery systems based on hyaluronic acid can bind to the inflamed areas of the colon (Liu et al., 2016; Vafaei et al., 2016; Kotla et al., 2019; Lee et al., 2020; Pan et al., 2020). Xiao et al. developed hyaluronic acid-functionalized nanoparticles loaded with Lysine-Proline-Valine tripeptide, an anti-inflammatory factor. Oral administration of the nanoparticles exhibited excellent accumulation of them in the damaged colon of DSS-induced colitis mice. Furthermore, the nanoparticles decreased signs related to colitis and facilitated mucosa healing (Xiao et al., 2017). One of the other attractive features of hyaluronic acid is that after breakdown, N-acetylglucosamine and Glucuronic acid act as building blocks for glycosaminoglycan synthesis and so accelerate bowel regeneration (Salvatore et al., 2000; Lee et al., 2020).

Besides, targeting the folate receptor can be regarded as an appropriate option for drug delivery to the injured colon because it was indicated that this receptor is overexpressed in the inflamed tissue (Naserifar et al., 2020). In one study, folate-functionalized PLGA/PLA nanoparticles loaded with 6-shogaol promoted the capacity of cellular uptake by Colon-26 cells through folate receptor-mediated endocytosis. 6-shogaol is a main component of dried ginger and has anti-inflammatory and antioxidative features. Oral administration of the nanoparticles considerably soothed colitis symptoms and facilitated bowel regeneration in DSS-treated mice *via* modulating the expression of anti-inflammatory (HO-1 and Nrf-2) and pro-inflammatory (TNF-α, IL-6, IL-1β, and iNOS) agents (Zhang et al., 2018). In a similar study, poly (amidoamine) dendrimers functionalized with folate accumulated in the inflamed colon of DSS-treated mice. These dendrimers are able to load drugs with high entrapment efficacy (Poh et al., 2017). Furthermore, some studies showed that folic acid supplements have a protective function in IBD-related colon cancer (Lashner et al., 1997; Chowers et al., 2000). From a mechanistic point of view, an inadequate supply of methyl group donors such as folate is associated with changed methylation of colonic DNA and the raise of colorectal carcinogenesis in rats, mice, and humans (Kim, 2005; Guruswamy et al., 2008).

**FIGURE 2:**
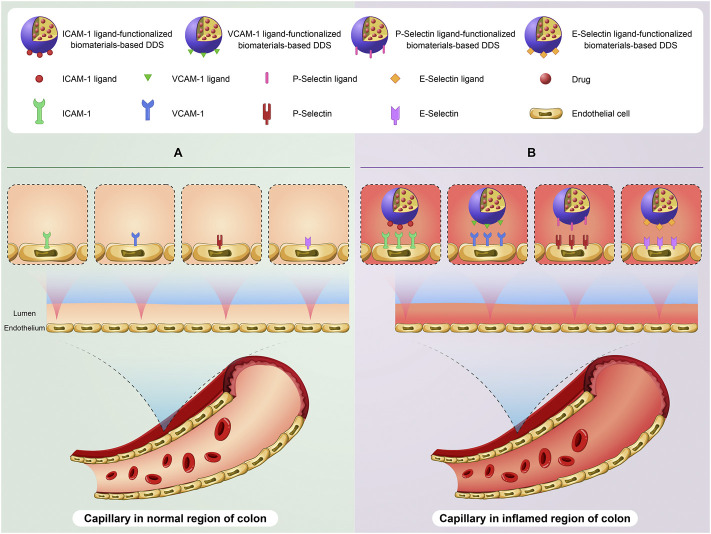
A schematic illustration of targeting expression changes of biomolecules on the surface of capillary endothelial cells in the colon of IBD patients using biomaterials-based drug delivery system (biomaterials-based DDS). The basic expression level of some biomolecules especially ICAM-1, VCAM-1, P-Selectin, and E-Selectin on the surface of capillary endothelial cells in the normal region of colon **(A)**. Targeting overexpression of these biomolecules using ligand-functionalized biomaterials-based DDS on the surface of capillary endothelial cells in the inflamed region of colon **(B)**.

On the other hand, the expression of some biomolecules on the surface of capillary endothelial cells raises in the inflamed colon. Consequently, targeting these changes *via* biomaterials-based DDSs functionalized with specific ligands of these biomolecules can be a promising option for delivery of drugs to the injured colon (Figure 2). For example, Sialyl-Lewis^x^ (a tetrasaccharide) is one of specific ligands of P-Selectin (Li C. et al., 2019). Eniola et al. developed PLGA microspheres functionalized with Sialyl-Lewis^x^. The results of adhesion assay demonstrated the great attachment of these microspheres to a slide coated with P-selectin (Eniola et al., 2002). In a similar research, IV injection of PLA–PEG microparticles conjugated to E-Selectin antibody showed excellent adhesion of them to inflamed endothelium in an *in vivo* model (Sakhalkar et al., 2003). It is important to point out that lymphocyte function-associated antigen 1 (LFA-1) is one of specific ligands of ICAM-1. LFA-1 is found on the surface of neutrophils and helps the specific adhesion of neutrophils to the surface of capillary endothelial cells in inflamed tissues *via* interaction with ICAM-1. Hence, inspired by interaction between neutrophils and capillary endothelial cells, the rational design of biomaterials-based DDSs through mimicking neutrophil membrane can be regarded as an promising candidate to deliver drugs to the inflamed colon. Interestingly, Zhao et al. developed neutrophils-simulated liposomes loaded with keratinocyte growth factor (KGF), which can help bowel regeneration and has a protective role against IBD. The uptake of these particles was successfully occurred by the inflamed human umbilical vein endothelial cells. Furthermore, the intravenous injection of these liposomes showed high accumulation of them in the inflamed colon of DSS-treated mice, leading to reduction of inflammation (Zhao et al., 2019).

In summary, targeting changes in the expression of biomolecules using biomaterials-based DDSs can be considered an appropriate option for drug delivery to the damaged colon provided that further studies on *in vivo* models are performed for the complete and accurate assessment of safety and efficiency.

## Challenges and future perspectives

Until now, numerous and interesting studies were performed to specifically target pathophysiological changes in IBD patients using biomaterials-based DDSs. However, there are still many challenges facing the development of these systems. For example, although pure pharmaceutical grade biomaterials are generally considered safe and non-cytotoxic, but when these biomaterials are used together with other reagents to form drug delivery systems, their pharmacokinetic properties may change, therefore, their biocompatibility level can alter. For this reason, a comprehensive evaluation regarding the safety of these systems should be taken into consideration in future studies (Su et al., 2019; Zhang et al., 2020).

On the other hand, from a pathophysiological point of view, UC and CD are multifactorial diseases with a combination of immunologic disturbances and genetic predisposition (Khor et al., 2011). For this reason, the designing and development of biomaterials-based DDSs *via* single targets may not be effective in the targeted treatment of both UC and CD in practice. Hence, development of versatile biomaterials-based DDSs with ability in the simultaneous targeting of multiple factors of UC and CD can be a promising option for more efficient treatment of IBD in future research (Cheng et al., 2021; Luo et al., 2021; Wang et al., 2021).

Furthormore, it is important to point out that the patients with IBD are susceptible to infection, especially *Clostridium difficile* infection. *Clostridium difficile* (a Gram-positive spore-forming bacterium) is considered one of the most important causes of healthcare-associated infections (Issa et al., 2007; Rodríguez et al., 2020; Boeriu et al., 2022; Sweeney et al., 2022). If there is a physical barrier that prevents the adhesion of some intestinal pathogens such as *Clostridium difficile* to inflamed colon epithelium, it can be a prophylaxis strategy for inflamed colon infection. Hydrogel-based enema as one of the most attractive biomaterials-based DDSs for local drug delivery can fulfill this aim because it adheres to the inflamed colon epithelium and forms a physical barrier (Hong et al., 2022). Hence, developing these enemas can help in the prophylaxis of inflamed colon infection.

## Conclusion

The most important pathophysiological changes at the inflamed colon include changes in pH, surface charge, the concentration of reactive oxygen species, and the expression of some biomolecules. Targeting pathophysiological changes in IBD patients using biomaterials-based DDSs is a promising option for the effective delivery of drugs to the injured colon. However, IBD is a multifactorial disease, so developing biomaterials-based DDSs through single targets may not be effective in the treatment of IBD. For this reason, the development of versatile biomaterials-based DDSs with ability in the simultaneous targeting of multiple agents of IBD may be regarded as a valuable and potential candidate for more efficient management of IBD in future studies.
